# Clay content and pH: soil characteristic associations with the persistent presence of chronic wasting disease in northern Illinois

**DOI:** 10.1038/s41598-017-18321-x

**Published:** 2017-12-22

**Authors:** Sheena J. Dorak, Michelle L. Green, Michelle M. Wander, Marilyn O. Ruiz, Michael G. Buhnerkempe, Ting Tian, Jan E. Novakofski, Nohra E. Mateus-Pinilla

**Affiliations:** 1 Illinois Natural History Survey – Prairie Research Institute, University of Illinois Urbana-Champaign, 1816 S Oak Street, Champaign, IL 61820 USA; 2 Department of Animal Sciences, University of Illinois Urbana-Champaign, 1503 S Maryland Drive, Urbana, IL 61801 USA; 3 Department of Natural Resources and Environmental Sciences, University of Illinois Urbana-Champaign, 1102 S Goodwin Ave, Urbana, IL 61801 USA; 4 Department of Pathobiology, University of Illinois Urbana-Champaign, 2001 S Lincoln Avenue, Urbana, IL 61802 USA

## Abstract

Environmental reservoirs are important to infectious disease transmission and persistence, but empirical analyses are relatively few. The natural environment is a reservoir for prions that cause chronic wasting disease (CWD) and influences the risk of transmission to susceptible cervids. Soil is one environmental component demonstrated to affect prion infectivity and persistence. Here we provide the first landscape predictive model for CWD based solely on soil characteristics. We built a boosted regression tree model to predict the probability of the persistent presence of CWD in a region of northern Illinois using CWD surveillance in deer and soils data. We evaluated the outcome for possible pathways by which soil characteristics may increase the probability of CWD transmission via environmental contamination. Soil clay content and pH were the most important predictive soil characteristics of the persistent presence of CWD. The results suggest that exposure to prions in the environment is greater where percent clay is less than 18% and soil pH is greater than 6.6. These characteristics could alter availability of prions immobilized in soil and contribute to the environmental risk factors involved in the epidemiological complexity of CWD infection in natural populations of white-tailed deer.

## Introduction

Soil is an important long-term reservoir for several pathogens associated with diseases including histoplasmosis, anthrax, and scrapie^1–3^. Understanding how soil properties affect disease transmission and persistence will strengthen disease surveillance and control efforts. Chronic wasting disease (CWD) is a disease for which soil has been shown to retain the infectious agent^4^. Like scrapie, CWD is a transmissible spongiform encephalopathy (TSE). It is caused by infectious prion proteins (disease-causing isoform PrP^sc^) that accumulate in several tissues including the central nervous system and cause slow neurodegenerative behavioral effects and changes in bodily functions in the infected host leading ultimately to death^5–7^. CWD is seen predominately in cervids including mule deer (*Odocoileus hemionus*), white-tailed deer (*Odocoileus virginianus*), elk (*Cervus elaphus*), and moose (*Alces alces*). Since its discovery in Colorado in 1967, CWD has been detected in wild cervid populations in 21 U.S. states, 2 Canadian provinces, and Norway^5,8,9^. In the U.S. state of Illinois, CWD was first reported in free ranging white-tailed deer in the northern part of the state in 2002 and has since been detected in 16 counties^10^. In response to the outbreak, the Illinois Department of Natural Resources (IDNR) designed and implemented a management strategy to lower deer densities in and around CWD affected areas to reduce the risk of disease transmission^11^. The strategy included surveillance of harvested deer throughout the state, reduction of deer densities in CWD affected counties through increased harvest opportunities, and localized culling in CWD infected areas including an approximate 2 mi^2^ buffer surrounding infected areas^10,12^.

CWD can be transmitted vertically (in utero from a mother to her offspring)^13,14^ or horizontally either directly or indirectly. Direct horizontal CWD transmission occurs via physical contact with prion infected animals, carcasses, body secretions and excreta^15,16^. Indirect CWD transmission between groups in a population occurs via ingestion and involves an inanimate vehicle (fomite, soil or plants) contaminated with infectious excreta, bodily fluids, antler velvet, and decaying carcasses of an infected host^15,17–24^. Airborne transmission of CWD was demonstrated in an indoor research facility following aerosol exposure of white-tailed deer to CWD infected brain homogenates^17^. The relative contribution of environmental contamination to the epidemiological dynamics of CWD remains an open question as the multifactorial nature of CWD is complex, and interactions between the three determinants of disease, the agent (infectious prion), the host, and the environment, are closely interconnected.

Empirical evidence suggests that soils are an environmental determinant of CWD influencing prion availability and persistence^25^. Adsorption, defined here as the physical or chemical binding of a prion to a soil solid phase, has been repeatedly examined for its role in prion persistence and availability in soil^26^. Many studies have shown the capacity of whole soils and mineral components to adsorb prions and have explored the mechanisms underlying prion-soil interactions^4,21,27,28^. Soil characteristics found to influence adsorption include texture (e.g. clay, sand, silt), organic matter (OM), soil moisture, pH, ionic strength, and cation exchange capacity (CEC)^27,29,30^. Because adsorption mechanisms and a soil’s capacity to adsorb vary depending on these characteristics, the amount of prion stabilized in soil also varies^25^. The current scientific ability to understand factors that determine whether prions immobilized in soils remain bioavailable to organisms that could degrade and/or ingest them is hindered by the absence of reliable experimental models and the complexity of these systems^27^. Yet an alternative option to improve our understanding of the role of soil characteristics on CWD are epidemiological observational cross-sectional studies based on existing real world data.

Modeling locations that are more likely to be associated with infection due to soil characteristics that enhance prion availability and persistence could improve our understanding of prion bioavailability in soils and help explain patterns of CWD persistence on the landscape. Such statistical models could become complementary tools to interfere with the transmission of CWD and thus reduce the morbidity and mortality associated with this disease. To date, predictive landscape models of CWD have been based primarily on landscape features related to deer habitat with limited input directly related to soil characteristics^31–34^. This study was designed to understand the contribution of soil characteristics to the epidemiology of CWD in deer using a predictive modeling approach. Based on existing data from CWD surveillance in Illinois deer, we identified chemical and physical soil characteristics in five counties where white-tailed deer have tested positive for CWD. We developed a predictive model of CWD on the Illinois landscape based on the association of CWD persistence and soil characteristics. Study objectives were to: 1) build a boosted regression tree (BRT) model using CWD presence/absence data and soil data to identify soil characteristics that have the greatest influence on the persistent presence of CWD, 2) assess the direction of effect of soil characteristics on the persistent presence of CWD, and 3) predict the probability of the persistent presence of CWD in northern Illinois based on soil characteristics.

## Results

### Model predictions

**Figure 1:**
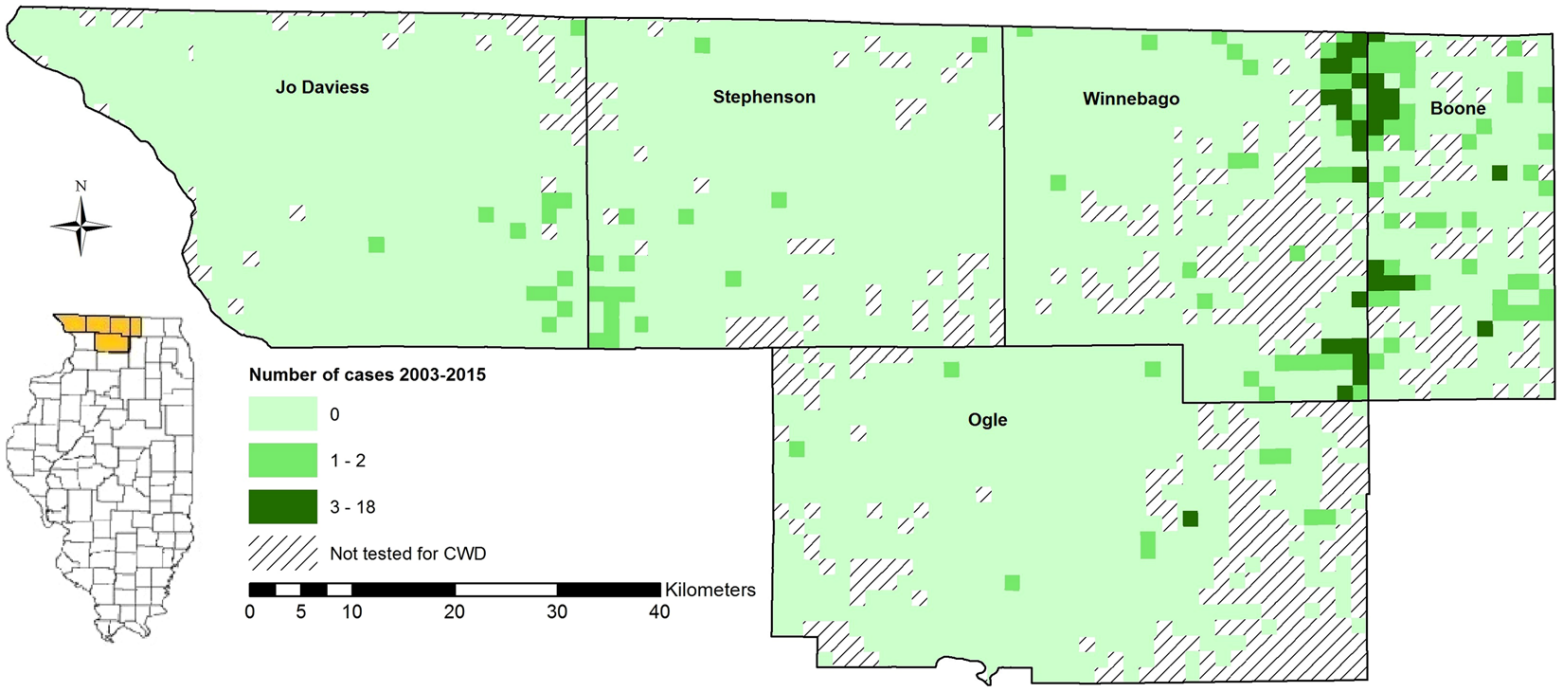
Tested TRS locations in five northern Illinois counties from 2003–2015. Color coded TRS locations had at least one deer tested for CWD from 2003-2015. Colors reflect the number of positive cases, and hashed TRSs were not tested for CWD. Map created using ArcMap 10.3 (ESRI, Redlands, California, USA).

**Figure 2:**
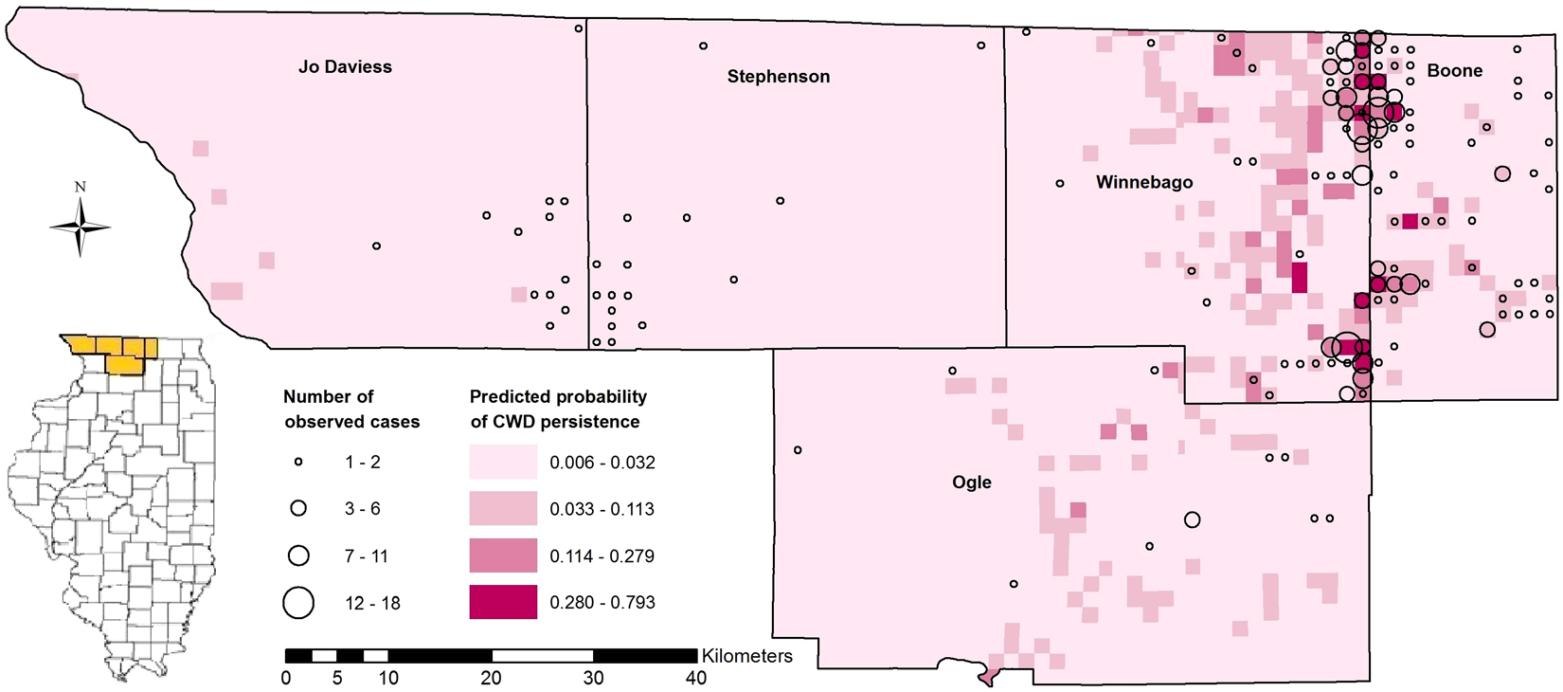
Predicted probability of CWD presence with the TRS locations of CWD-positive cases from 2003–2015. Predicted probabilities increase as colors progress from light to dark. Graduated circles indicate the number of observed CWD-positive deer in each TRS. Map created using ArcMap 10.3 (ESRI, Redlands, California, USA).

Our BRT model sought to identify relationships between soil characteristics (clay, sand, silt, OM, water content, pH, and CEC, Table 1) and the persistent presence of CWD. We defined “persistent presence” as a township-range-section (TRS – 1 mi^2^ grid specified by Public Land Survey System) with ≥3 deer testing positive to CWD (Fig. 1). The BRT model predicted the probability of the persistent presence of CWD in each TRS within the test dataset with an area under the receiver operator characteristic curve (AUC) score of 0.944, where an AUC score of 1.0 represents a perfect prediction, and a score of 0.5 is equivalent to random guessing. The maximum kappa score, 0.69, (a kappa of 1.0 is perfect agreement between predicted and observed locations and a kappa of 0.0 represents chance agreement), was achieved with a threshold probability of 0.19 that delineated CWD presence from absence. Our model accuracy was significantly better than the No Information Rate (p = 0.0008). Predicted probabilities ranged from 0.006 to 0.793 across all TRSs (Fig. 2). The highest predicted probabilities were located in Boone and Winnebago counties. Moderate predicted probabilities, ranging from 0.033 to 0.096, were predicted along the western and southern edges of Jo Daviess County. Stephenson County, in general, had the lowest predicted probabilities with all TRSs predicting ≤0.032. Although the majority of Ogle County had very low predicted probabilities (≤0.032), several TRSs in the central and eastern portions of the county had somewhat higher predicted probabilities ranging up to 0.191.

**Table 1:** Soil-prion/protein interactions.

Soil characteristic	Interaction with prions/proteins	Average in study area (range)	Average in model (range)
Clay	Binds strongly to prions, affects availability of prions^4,35,37^	21.8% (4.8–29.3%)	21.7% (4.8–28.7%)
Sand	Binds less with prions relative to silt and clay^27,35,39^	13.7% (2.8–87.5%)	13.3% (3.0–87.5%)
Silt	Binds less with prions relative to clay^66^	64.4% (7.7–78.3%)	64.9% (7.7–77.4%)
Organic matter	Binds with prions, affects availability^21,48^	3.1% (1.1–23.4%)	3.0% (1.1–23.4%)
Water content	Affects decomposition of proteins^43,67^	29.2% (13.3–35.7%)	29.1% (13.3–35.7%)
pH	Affects prion charge and adsorption/desorption to soil particles^27,29,42,43^	6.5 (5.8–7.7)	6.5 (5.8–7.3)
Cation exchange capacity (CEC)	Affects binding to soil particles^68^	18.4 (5.3–50.0)	18.3 (5.3–50.0)

Soil characteristics and their corresponding interaction with prions/proteins and observed ranges and averages in the study area and in the model dataset.

### Effect of soils on the predicted probability of the persistent presence of CWD

**Figure 3:**
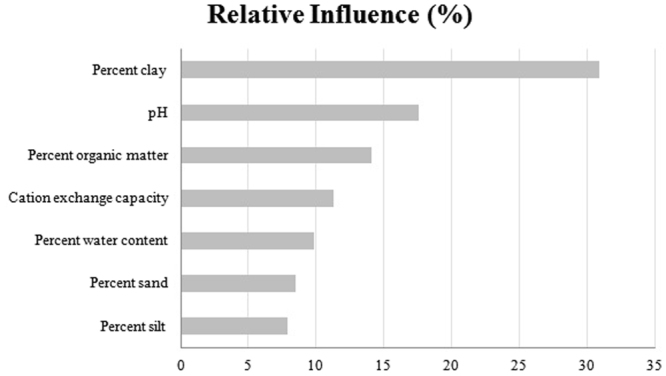
Relative influence of soil characteristics on the persistent presence of CWD. The relative influence is a scaled value that describes the contribution of each of soil characteristic to the prediction of the persistent presence of CWD based on the number of times a variable is used as a predictor in the model weighted by the improvement in model fit due to inclusion^50^; see Methods.

**Figure 4:**
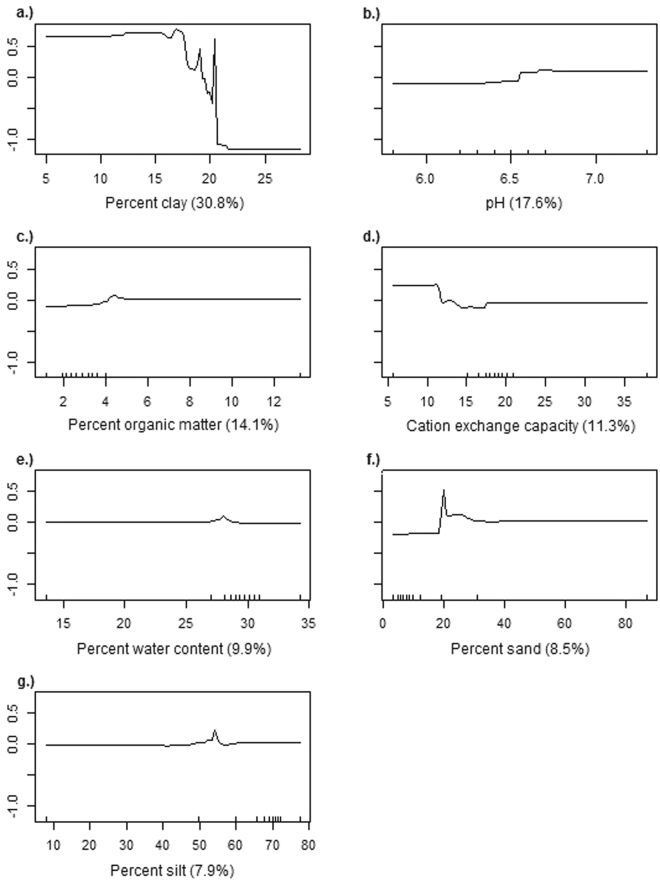
Partial dependence plots of soil characteristics with the relative influence (%). The x-axis represents the values associated with the soil characteristic in each TRS. The y-axis represents the effect of the soil characteristic on the probability of the persistent presence of CWD where positive values indicate a positive effect and negative values indicate a negative effect. Tick marks along the x-axis indicate observed deciles of each variable.

**Figure 5:**
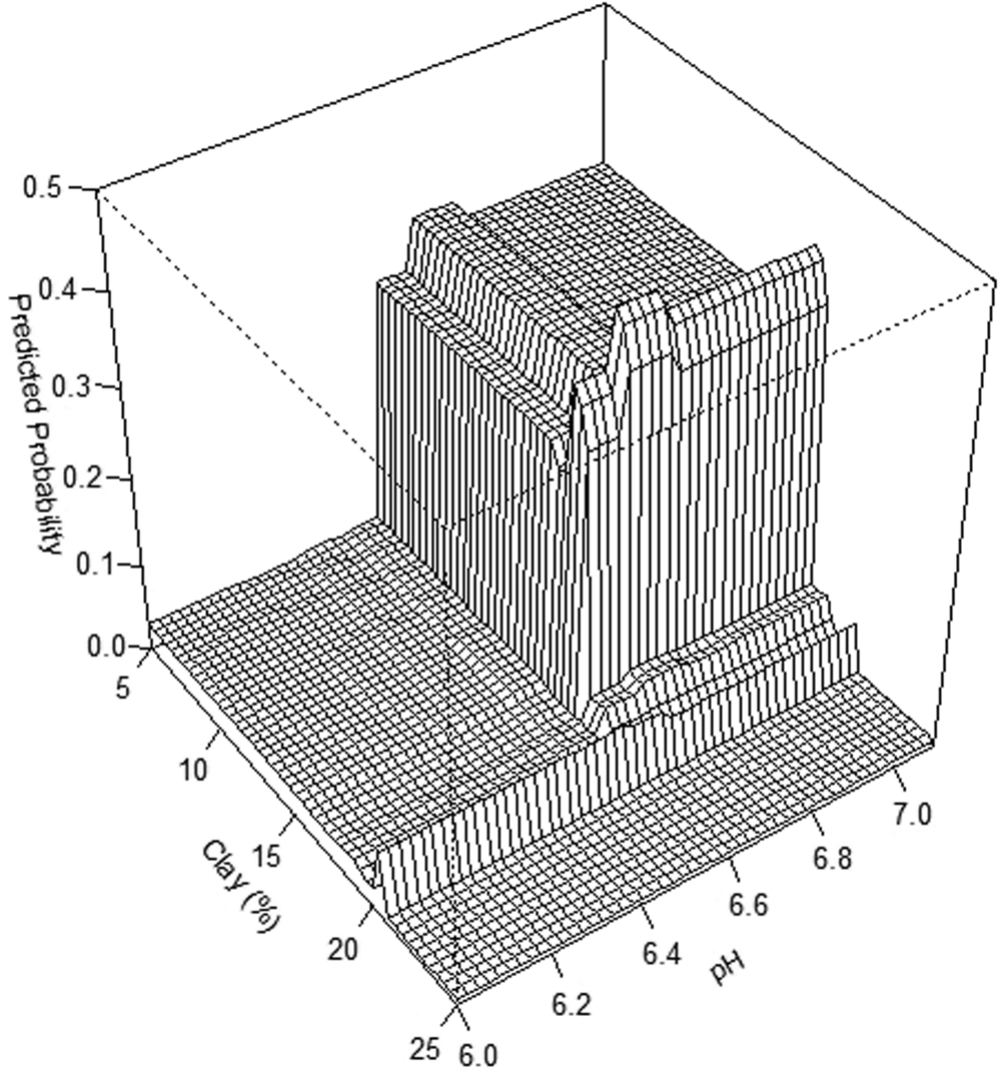
Three dimensional partial dependence plot of interaction between percent clay and pH. Shows the model predicted probability of the persistent presence of CWD for combinations of percent clay and pH while averaging over all other predictors.

The model indicated that the two most important predictors based on their relative influence (i.e., a scaled percentage value that describes the contribution of each variable to predicting the persistent presence of CWD in the BRT model) were percent clay (30.8%) and pH (17.6%) (Fig. 3). Partial dependence plots illustrated the effect of each soil characteristic on the probability of the persistent presence of CWD (Fig. 4). The results demonstrated that when the percentage of clay exceeded approximately 18%, the predicted probability decreased significantly (Fig. 4a). Below a pH of 6.6, the predicted probability was low, whereas above a mean pH of 6.6, the predicted probability increased (Fig. 4b). The strongest interaction effect was observed between clay and pH. Here, the predicted probability of the persistent presence of CWD due to clay was affected by pH when clay was below 18%. Once clay reached and exceeded 18%, the predicted probability was low regardless of pH (Fig. 5).

According to a map of majority land cover types in our study area (Supplementary Figure 1), the majority of TRSs can be divided into the following five TRS groups: open water, developed space, forest, cultivated crops, and herbaceous. The distribution of soil characteristics for each of those five land cover types was quite similar, and the range of soil characteristics observed was similar for each TRS group, especially for our most influential predictors, percent clay and pH. In addition, to reduce observational bias based on spatial variation of hunting and culling efforts, we ensured that hunting and culling efforts had occurred throughout the study area. The large distribution of tested TRSs across years in our study reduced observational bias.

## Discussion

CWD is an infectious disease caused by a transmissible prion protein (PrP^sc^) that replicates in the affected host. Infection occurs when the susceptible host is exposed to and acquires the PrP^sc^. The PrP^sc^ can be acquired from the environment or from other hosts that harbor CWD. Transmission from one host to the other leads to temporal and spatial chains of transmission through the host population. Although this study does not account for the complex and multifactorial interactions between the characteristics of the deer population, the agent, and the environment, this analysis suggests that soil characteristics are associated with the persistent presence of CWD in the study area.

Soils may serve as a complex reservoir by either enhancing or inhibiting prion availability. The model suggests that clay content has the strongest influence on the persistent presence of CWD. Clay has been associated with CWD in this and many other studies although its effect on infectivity is debated^21,32,35–37^. The clay fraction disproportionately influences soils’ chemical interactions because small particles found in this size range (<0.002 mm) have a relatively large surface area to interact with ions in soil solution and have greater cation exchange capacity (3–150 meq/100 g) than minerals found in the silt and sand sized fractions^38^. In this model, the probability of the persistent presence of CWD is greater at lower percentages of clay. The model indicates a threshold value for clay content at approximately 18%. The model indicates higher predicted probabilities of the persistent presence of CWD when clay content is less than 18% and lower predicted probabilities when clay content is greater than 18%. This threshold may seem at odds with some previous studies that suggest prion disease incidence increases with clay abundance due to increasing prion immobilization and persistence; however, those works have been carried out in regions where average clay contents are lower than average clay contents found in this study^25^. Soils with clay contents above 18% may reflect reductions in prion bioavailability due to immobilization where clay levels are higher. Interactions between prions and clay may be affected by pH, ionic strength, and the specific ions present^39,40^. Though our model is in agreement with a broader study in the Midwest where more clay is associated with less CWD^31^, the effect of clay seen in this study is the opposite of results modeled for clay in Colorado which showed that for every 1% increase in clay, there was an 8.9% increase in CWD in deer^32^. Many soils in northern Colorado have a higher electrical conductivity (a measure of the salinity of soil) than are found in northern Illinois^41^. In Colorado, the increased concentration of salts may compete for binding sites on the surface of clays preventing the adsorption of prions and increasing the concentration of unbound prions in the soil. This is consistent with an elevated pH that would promote desorption in the same way predicted by our model.

Soil pH effects are likely associated with changes in the adsorption behavior of prions. Shifts in pH can alter prion size and degree of aggregation, surface charge of soil minerals and organic matter, and the size of the soil-water interface that controls adsorption-desorption behaviors^27,29,42,43^. The distinct threshold in the influence of pH on the persistent presence of CWD is notable and suggests an isoelectric point (IEP), the pH at which the prion has a net zero charge, around pH 6.6. This value falls within the IEP range (pH 4.6–7.9) reported for prion proteins^27,44^. If the IEP for the pathogenic prion is at pH 6.6, then it would have a net positive charge associated with the N-terminal end at and below this pH and would therefore be attracted and bind to negatively charged soil and organic matter surfaces. Above pH 6.6, the PrP^sc^ would have a net negative charge and be repelled by negatively charged surfaces such as clay and organic matter. This could leave the prion in solution where it would be more bioavailable and mobile within the environment. Increased bioavailability of unbound prions to plants could also increase the potential for further disease transmission especially if prion uptake occurred in vegetation that was commonly foraged by deer. Desorption of prions affiliated through a variety of mechanisms can be induced at high pH^27,45^.

Our results indicate that the physical mechanism of immobilization on the solid phase and remobilization control prion availability. When immobilization is reduced (below 18% clay or above pH 6.6), we see an increase in the probability of the persistent presence of CWD. Reduced immobilization results in increased movement of unbound agents and dispersal in aqueous forms. Lower abundances of clay also result in the reduced physical stability of soils and increased susceptibility to wind erosion and dust emissions^29,46^. Prions have been detected in dust^47^, and if deer can acquire CWD from dust-bound prions, it can be surmised that soil characteristics and conditions that favor dust emissions such as less physically stable soils are more likely to be aerosolized during grazing or rutting behaviors potentially leading to enhanced exposure of deer to prions in the soil through inhalation and ingestion of dust-bound prions.

By focusing on the “persistent presence” of CWD and defining it as ≥3 deer cases in a TRS, we recognize that the model could be missing newly emerging areas. For example, the model predicted very low probabilities in a cluster of TRSs where one or two CWD cases were reported along the southern edge of the Jo Daviess – Stephenson county line. The model could be under predicting this region because TRSs containing only one or two CWD cases between 2003 and 2015 were omitted from the model dataset, and the soil characteristics present here may not be typical of those that support the persistent presence of CWD in other parts of the region. To assess this possibility, we developed an alternative model in which TRSs with all cases were included in the dataset and classified as present (Supplementary Figs 2–4). These model results indicated that the most influential predictors were percent clay and pH, the same as our result for our model where persistent presence is defined as ≥3 CWD cases. However, the model in which TRSs with all cases were included produced relatively lower Kappa statistics and higher prediction error. Because we wanted to increase the opportunity to see environmental persistence of the disease, reduce the chance of transient deer affecting the disease status of a TRS, and because of the less accurate predictive capabilities, we did not consider TRSs with 1 or 2 cases in our BRT model. It could also be interpreted that the soil characteristics in this particular area do not favor reservoir development by making the infectious prion unavailable or by having characteristics that speed degradation of the prion. Areas in which CWD cases have been detected but predicted probabilities are low based on soil models may be important during disease management efforts because control by culling may be more effective in areas were environmental conditions are unfavorable for CWD transmission and persistence. It is important that management efforts focus on high risk areas for disease transmission, but the type of control can be more effective when matched with likely pathways of transmission.

This model indicates that soil characteristics can be an important piece of information to help when predicting the probability of CWD persistence on the landscape. The model highlighted the importance of the presence and abundance of soil properties associated with the availability and persistence of infectious prions in the environment. It also helps to infer mechanisms responsible for these differences–particularly when considered alongside studies in other areas where soils are a potential reservoir. Mixed findings in the literature have cited the complexities of soil factors and associated mechanisms driving prion sorption to soils, leaving us with limited understanding of how the infectivity and availability of prions might be affected across soil characteristics and geographical landscapes^4,21,28,35,48,49^. However, based on the interactions between proteins and soils, our model indicates that the persistent presence of CWD on the landscape might be predictable based on soil characteristics suggesting that it can be used in formulating CWD control management strategies.

This study provided novel observational insights into field conditions conducive to the development of a CWD reservoir in the environment. Even though the predictions derived from this model were developed through correlative analysis, they can be used to initiate new surveillance areas and management tactics by identifying TRSs where the predicted probability of the persistent presence of CWD is elevated. Areas with higher deer densities that overlap TRSs with higher predicted probabilities of the persistent presence of CWD due to soil characteristics could guide management officials to intensify density reduction efforts to limit infected deer and their contribution of prions shed into the environment. If travel corridors connect TRSs where CWD is known to occur to TRSs where the predicted probability is higher, CWD control management strategies have a rationale to reduce deer movement between those areas.

BRT models are valuable for predicting areas prone to CWD persistence, offering a practical tool for managers to use when delineating and prioritizing disease management areas. Although BRT models are complex, ample literature and working guides^50–54^ exist to help navigate the process, and the software is freely available. Predictive models can improve our understanding of CWD by revealing gaps in knowledge that point to future studies. This study focuses specifically on the evaluation of associations between soil characteristics and the persistent presence of CWD on the landscape and provides additional value by highlighting contributing factors to CWD reservoir development and environmental transmission. Observational epidemiological studies in natural settings are exceedingly complex, and CWD is at the center of a system influenced by a wide range of variables surrounding deer density, behavior, and habitat use.

We recognize that factors besides soils characteristics related to the infectious prion protein, the environment, and white-tailed deer in Illinois serve as determinants of CWD transmission. Some of these factors include direct contact with a CWD infected host, deer densities, contact rates^55^, deer demographics^32,56^, genetic susceptibility^57^, or landscape characteristics (vegetation, elevation, slope, distance to riparian, etc.)^31,32,34^ that may affect deer movement and population dynamics. In Illinois, the home range size for resident white-tailed deer is 99 ha^58^. Although home ranges differ depending on factors such as sex and season, this value is within the size of the typical TRS, roughly 259 ha. However, this study did not evaluate deer movement as a risk factor for CWD. Neither did we evaluate the contribution of other risk factors in the spread and transmission of CWD in free ranging white-tailed deer populations such as the associations between soil characteristics and landscape characteristics (e.g., agriculture, vegetation, and bodies of water) or their effect on deer behavior. However, we did not find a strong association between soil characteristics and landscape characteristics, i.e. the pattern of soil characteristics did not vary across each of type of landscape. We did not evaluate the impact of areas where deer contact rates increase by human activities (e.g., captive deer facilities and supplemental feeding stations).

A broad epidemiological model evaluating environmental factors (other than soil characteristics) influencing indirect and direct transmission of CWD remains vital for the understanding of the transmission dynamics of CWD in free ranging white-tailed deer. Therefore, as a follow up to this work, we suggest a comprehensive model combining soil characteristics, landscape environmental features, contact rates and host-protein interactions to improve the predictive probabilities of CWD (transmission and spread) over the landscape, and its persistence in fomites and the environment. Nevertheless, the results from this study enhance knowledge of prion-soil interactions and serve as a guide to future studies.

## Methods

### Study area

A region in northern Illinois comprised of Jo Daviess, Stephenson, Winnebago, Ogle, and Boone counties was chosen as the study area based on the availability of data from a long history of CWD testing. This five-county region is bordered by the Mississippi River to the west, contains the Rock, Pecatonica, and Kishwaukee Rivers as well as many smaller waterways, and has a mixture of cropland, pastureland, developed lands, deciduous forest, grasslands, and wetlands (Supplementary Figure 1)^59^. CWD was first detected in Illinois along the Boone-Winnebago county border, and CWD-positive deer have been consistently identified in these two counties since 2002 (n = 316). Jo Daviess and Stephenson counties are situated to the west of Winnebago and have had a small number of CWD cases (n = 50) with the first detected case in 2008. Ogle County, directly south of Winnebago and Stephenson counties, has had relatively few cases of CWD (n = 21) with the first case detected in 2006.

### CWD data

IDNR conducts CWD surveillance of harvested deer and performs localized culling of deer in areas where CWD is known to occur to reduce disease transmission through a reduction in deer density. Deer samples were taken from a number of harvest sources including hunter harvest, sharpshooting, and culling of suspect deer. Although deer samples were tested from suspect deer (sick or road-killed), these samples were not included in the analysis. Tissue samples (retropharyngeal lymph nodes and obex) collected from deer harvested in the five-county region were tested by the Department of Agriculture Illinois Animal Disease laboratories for the presence of CWD using the gold standard immunohistochemical examination. Based on the immunohistochemistry test results for each sample, a disease status of positive or not detected was specified. The location of each sample was recorded as the township, range, section number in which it was collected as defined by the Public Land Survey System (PLSS) referred to in this study as TRS. The CWD data were summarized by TRS in order to classify CWD persistence at the TRS level. TRSs (n = 2235) included in the dataset used to build and test the model had at least one deer tested for CWD between 2003 and 2015 (Fig. 2). We defined TRS-level CWD persistence as a TRS that contained three or more CWD positive deer detected between 2003 and 2015 (n = 33) so as to ensure that infected deer had repeatedly been detected in a TRS (indicating disease persistence and the potential development of a soil reservoir) thus minimizing the chance of false positives for modeling purposes. We defined absence as a TRS that contained zero cases of CWD over the 13-year period (n = 2202) (Fig. 3). Because TRSs with one or two CWD positive deer may represent either transient occupation by infected deer or initial emergence of CWD in the TRS, these were not included in the analysis, thereby reducing the likelihood of false negatives. An alternative model was also developed to assess the differences in model performance with different definitions of TRS-level CWD presence (Supplementary Information). In the alternative model, we defined TRS-level presence as a TRS that contained at least one CWD positive deer and absence as a TRS that contained zero cases of CWD.

### Soil data

We used the United States Department of Agriculture Natural Resource Conservation Service (NRCS) Soil Data Viewer in conjunction with ArcMap 10.3 (Environmental Systems Research Institute [ESRI], Redlands, California, USA) to access and map soil type characteristics from the Soil Survey Geographic database v. 2.3.3^60^. From the soils database, we converted seven soil characteristics of the surface layer of soil (top five centimeters) with a weighted mean aggregation method to raster at 30 m resolution using the NAD 1983 UTM Zone 16 N coordinate system. We used zonal statistics to calculate the mean pH, percent clay, percent silt, percent sand, percent OM, CEC, and the percent water content at field capacity for each TRS. These soil characteristics were selected as predictors of CWD persistence based on their association with interactions between soils and proteins/prions. Soil maps delineate each soil type on the landscape where polygons represent an area determined to have relatively homogenous soil characteristics and topography. Most TRSs contained multiple soil polygons; therefore, an area-weighted average was used to estimate the mean of each soil characteristic in each TRS. The range and average of each soil characteristic in the study area were calculated (Table 1). We used hierarchical clustering to assess whether soil characteristics varied across land cover types.

### Boosted regression tree model to predict the persistent presence of CWD

To assess the relationship between soil characteristics and CWD persistence in a TRS, we created a boosted regression tree (BRT) model which uses a combination of boosting, a technique used to improve model accuracy by combining many simple models iteratively to reduce predictive error, and regression trees, models in which repeated binary splits in predictors classify a response^50,53^. Three main meta-parameters are used to maximize predictive performance and reduce overfitting: bag fraction, tree complexity, and learning rate^53^. The bag fraction represents the proportion of the data used to fit the model in each step. Tree complexity defines the number of nodes in each tree and represents the maximum order of interactions that can be fitted. Learning rate (or shrinkage rate) determines the contribution of each tree to the model as it grows. After systematically testing different combinations of settings for these parameters, the predictive performance of this model was maximized when we used a bag fraction of 0.75, a tree complexity of 5, and a learning rate of 0.001. The BRT model was fit using 80% of the data (n = 1788), and the model predictions were validated with the remaining 20% (n = 447). The predictive ability of the model was determined based on the area under the receiver operator characteristic curve (AUC). The area under a plot of the true positive rate versus the false positive rate assessed at various classification thresholds (in this case, values of the predicted probability of CWD persistence) represented the model’s ability to distinguish between presence and absence^61^. We calculated kappa scores to assess the level of agreement between the model predictions of case locations and the observed cases of disease on the landscape and used the predicted probability of CWD persistence at which kappa was maximized as the classification threshold for determining the CWD status of a TRS^62^. To assess whether our model accuracy was better than the No Information Rate or a simple majority rules prediction, we created a confusion matrix. We then applied the model to all of the TRSs (n = 2829) within the study area to obtain model predictions for the entire region. We used the statistical program R and packages ‘dismo’, ‘gbm’, and ‘caret’ to perform all analyses^51,54,63,64^.

### Determining the influence of soils

The relative influence of the soil characteristics on CWD persistence was estimated as a part of the package ‘gbm’ in R^54^. The values for relative influence represent the average across all trees of the number of times a soil characteristic was selected for splitting within a tree weighted by the improvement made on the model as a result of that split^50^. These values are then transformed so that each assigned value reflects a percentage of contribution to the response. To determine the direction of the effects of each soil characteristic, we generated partial dependence plots for each soil characteristic on the probability of CWD persistence. The partial dependence plots serve as visual guides for interpreting how each soil characteristic influences the model predictions after the effects of all other soil characteristics have been averaged out^65^. Finally, we assessed the relative strength of interactions present between soil characteristics (or the relative contribution of the interaction between two soil characteristics to the predictive performance of the model) and generated a three-dimensional partial dependence plot of the strongest interaction^50,53^.

### Data availability

The data that support the findings of this study are available on request from the corresponding author (N.E.M.P.).

## Electronic supplementary material

Supplementary Information
