# Supplementary material for: Clay content and pH: soil characteristic associations with the persistent presence of chronic wasting disease in northern Illinois
Source: Sci Rep. 2017 Dec 22;7:18062. doi: 10.1038/s41598-017-18321-x (PMC5741720; doi:10.1038/s41598-017-18321-x)
Supplement: Supplementary file 1 — Supplementary Information [file 41598_2017_18321_MOESM1_ESM.pdf]

Clay content and pH: soil characteristic associations with the persistent presence of chronic wasting disease in northern Illinois

Sheena J. Dorak[1], Michelle L. Green[1,2], Michelle M. Wander[3], Marilyn O. Ruiz[4], Michael G. Buhnerkempe[1], Ting Tian[1], Jan E. Novakofski[2] & *Nohra E. Mateus-Pinilla[1]

[1]Illinois Natural History Survey – Prairie Research Institute, University of Illinois Urbana-Champaign, 1816 S. Oak Street, Champaign, IL 61820, USA.

[2]Department of Animal Sciences, University of Illinois Urbana-Champaign, 1503 S. Maryland Drive, Urbana, IL 61801, USA.

[3]Department of Natural Resources and Environmental Sciences, University of Illinois Urbana-Champaign, 1102 South Goodwin Ave, Urbana, IL 61801, USA.

[4]Department of Pathobiology, University of Illinois Urbana-Champaign, 2001 South Lincoln Avenue, Urbana, IL 61802, USA.

Supplementary Information

Supplementary Figure S1. Majority land cover type in each TRS. Color coded majority land cover type according to 2011 National Land Cover Database[1] for each TRS in the study area with larger rivers and streams given as visual reference. Map created using ArcMap 10.3 (ESRI, Redlands, California, USA).

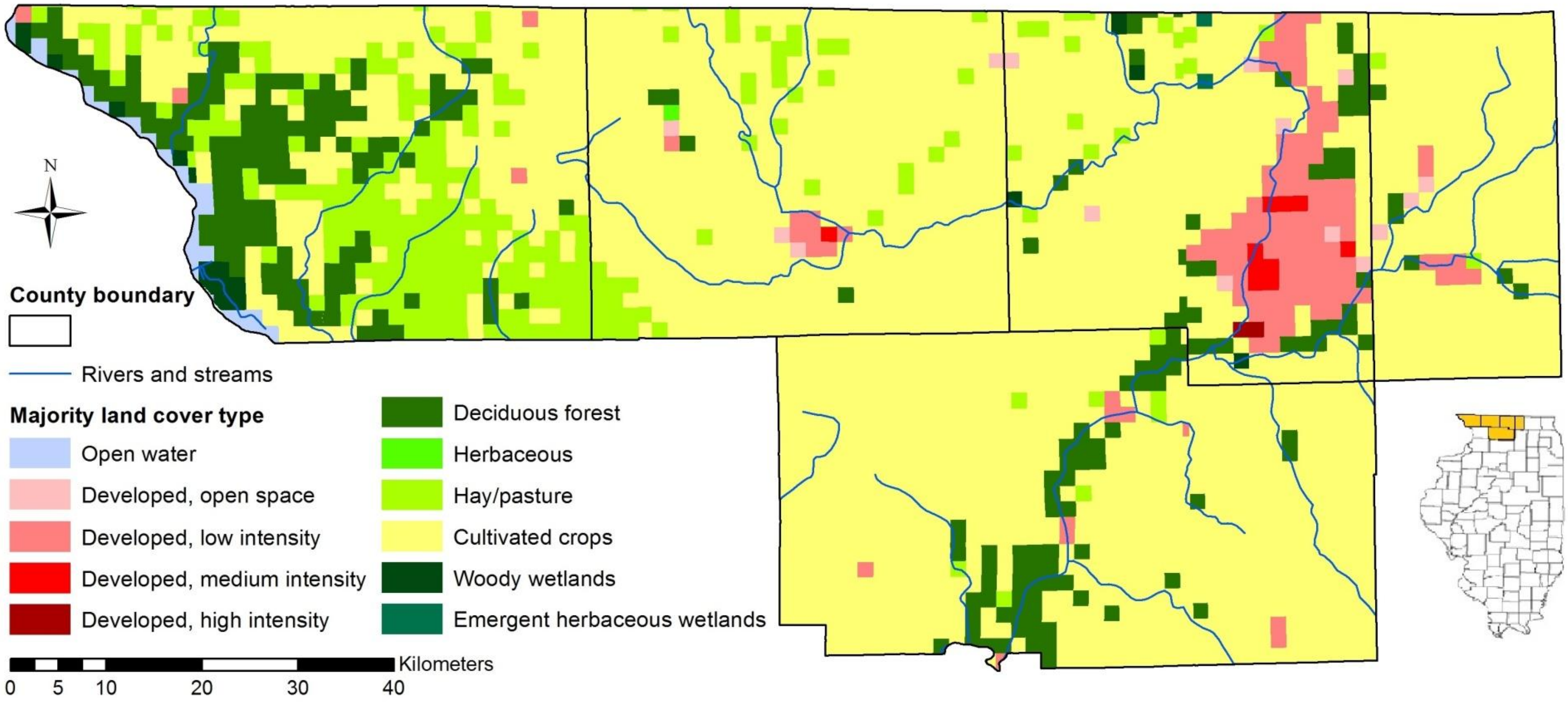

Alternative boosted regression tree model

We developed a model in which TRSs with ≥ 1 CWD case were included in the dataset and classified as present (n = 141) and absence was defined as tested TRSs with no cases detected (n = 2206). The BRT model predicted the probability of CWD presence in each TRS within the test dataset with an area under the receiver operator characteristic curve (AUC) score of 0.785. The maximum kappa score, 0.38, was achieved with a threshold probability of 0.16 that delineated CWD presence from absence. Predicted probabilities of CWD presence ranged from 0.036 to 0.642 across all TRSs (Supplementary Fig. S2). The highest predicted probabilities were located in Boone and Winnebago counties. Moderate values of the probability of CWD presence, ranging from 0.036 to 0.127, were predicted along the western and southern edges of Jo Daviess County. Stephenson County, in general, had the lowest predicted probability of CWD presence with all TRSs predicted ≤ 0.06. Although the majority of Ogle County had a very low predicted probability of CWD presence (≤ 0.06), several TRSs had somewhat higher predicted probabilities ranging up to 0.271. The model indicated that the two most important predictors based on their relative influence were percent clay (27.1%) and pH (20.5%) (Supplementary Fig. S3). Partial dependence plots illustrated the effect of each soil characteristic on the probability of CWD presence (Supplementary Fig. S4). The results showed when the percentage of clay exceeded approximately 20%, the predicted probability of CWD presence decreased significantly (Supplementary Fig. S4a). Below a pH of 6.6, the probability of CWD presence was low, whereas above a mean pH of 6.6, the predicted probability of CWD presence increased (Supplementary Fig. S4b).

Supplementary Figure S2. Predicted probability of CWD presence with the TRS locations of CWD-positive cases from 2003-2015. Predicted probabilities increase as colors progress from light to dark. Graduated circles indicate the number of observed CWD-positive deer in each TRS. Map created using ArcMap 10.3 (ESRI, Redlands, California, USA).

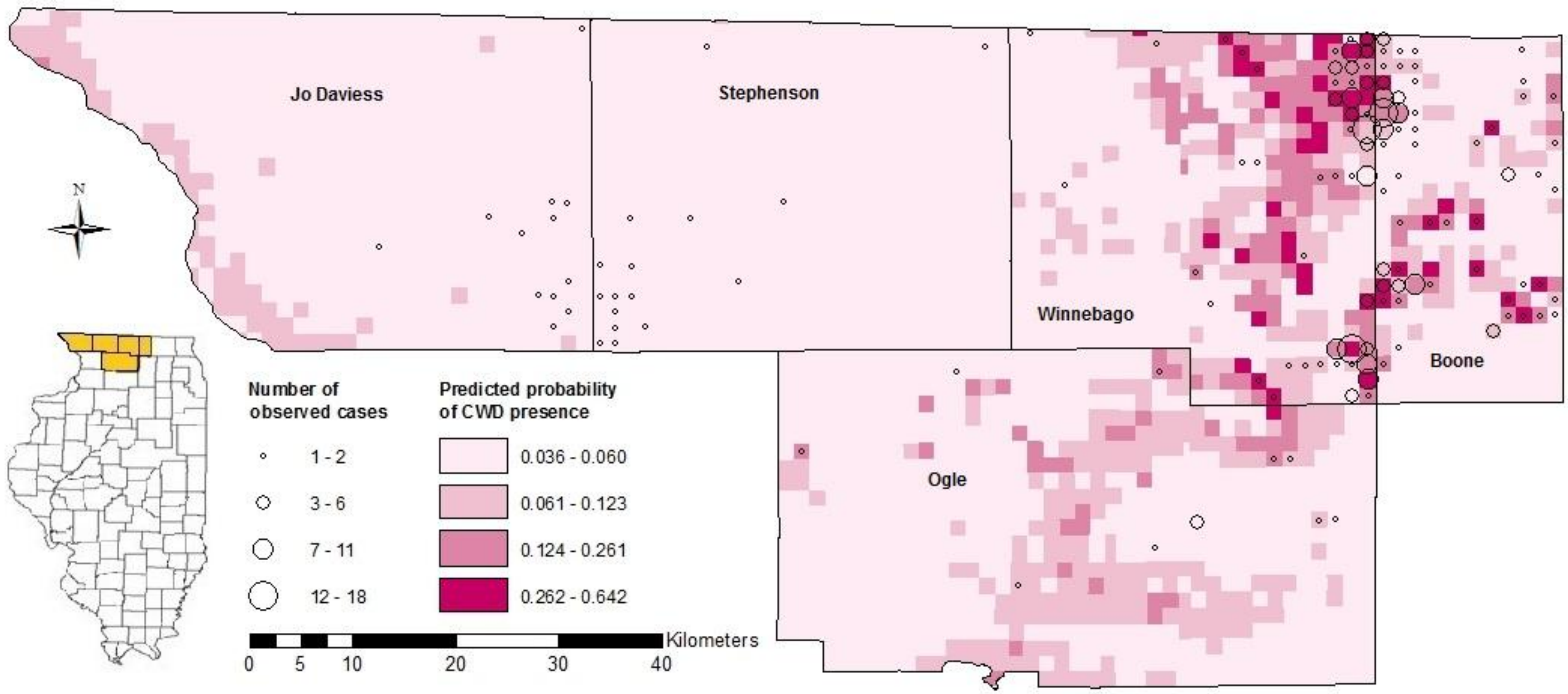

Supplementary Figure S3. Relative influence of soil characteristics on CWD presence. The relative influence is a scaled value that describes the contribution of each of soil characteristic to the prediction of CWD presence based on the number of times a variable is used as a predictor in the model weighted by the improvement in model fit due to inclusion[2].

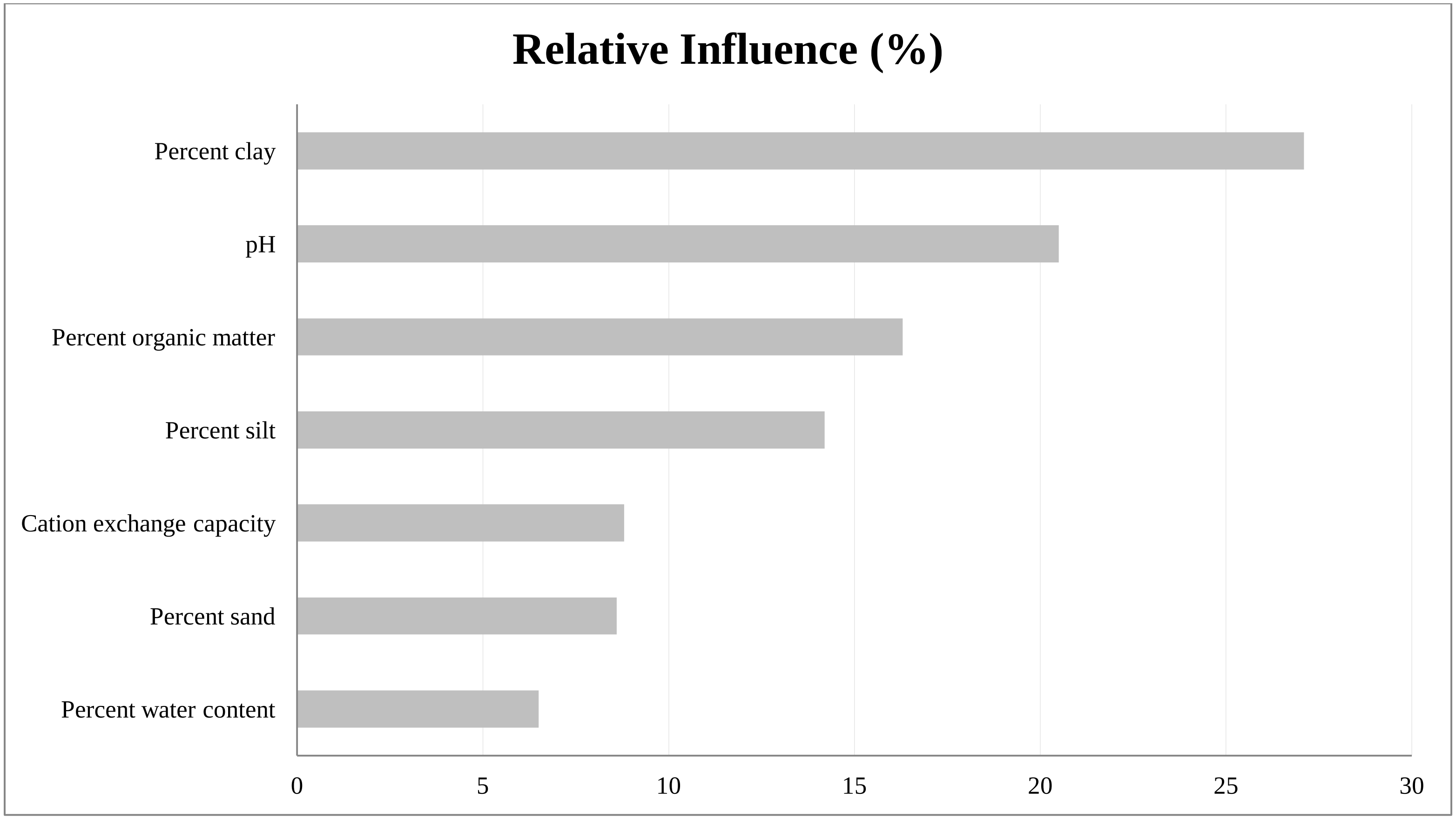

Supplementary Figure S4. Partial dependence plots of soil characteristics with the relative influence (%). The x-axis represents the values associated with the soil characteristic in each TRS. The y-axis represents the effect of the soil characteristic on the probability of CWD presence where positive values indicate a positive effect and negative values indicate a negative effect. Tick marks along the x-axis indicate observed deciles of each x variable.

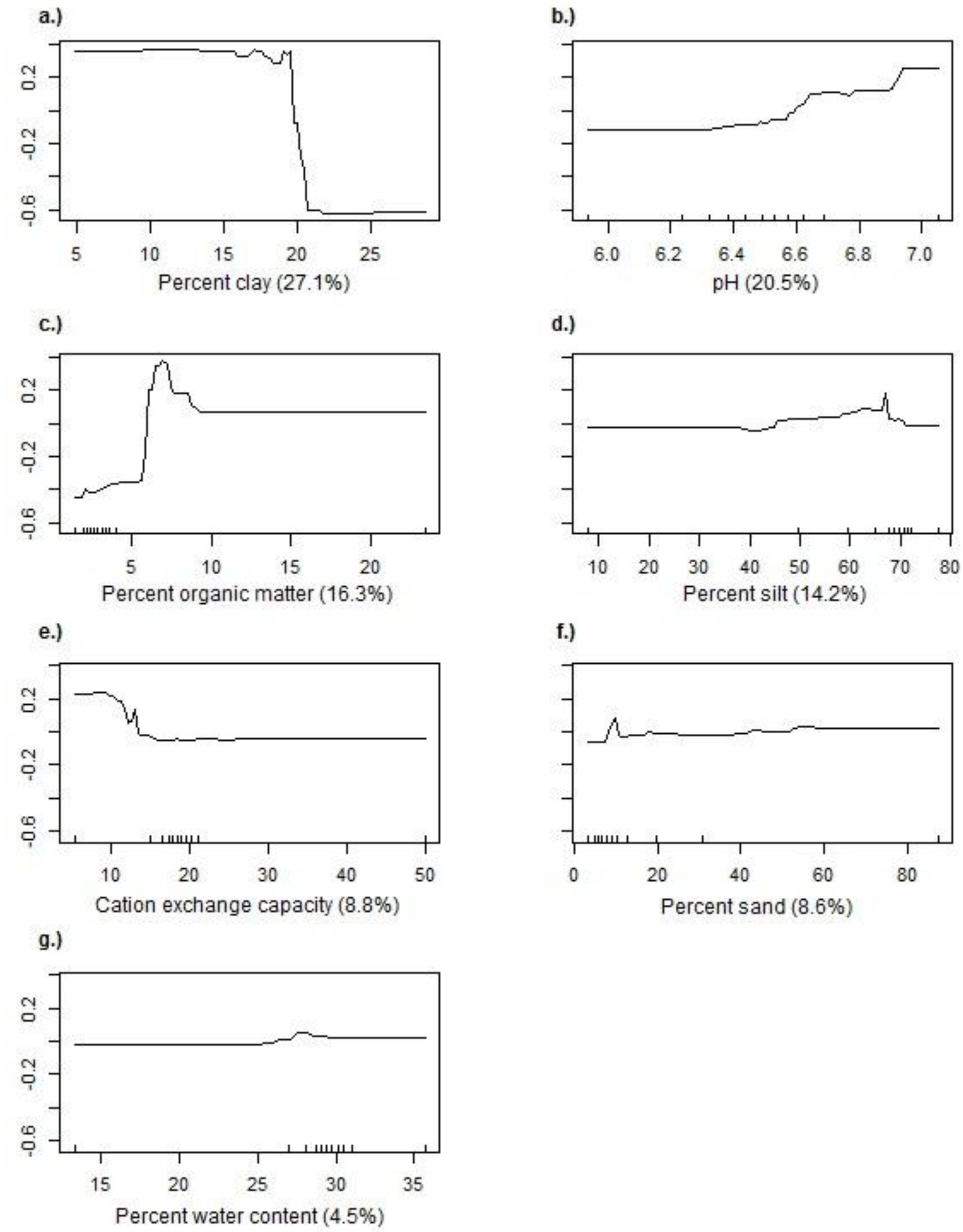

References

1. Homer, C. G. *et al.* Completion of the 2011 National Land Cover Database for the conterminous United States-Representing a decade of land cover change information. *Photogramm. Eng. Remote Sensing* **81,** 345–354 (2015).

2. Elith, J., Leathwick, J. R. & Hastie, T. A working guide to boosted regression trees. *J. Anim. Ecol.* **77,** 802–813 (2008).
